# GDF15-integrated blood biomarker panel for risk stratification of digestive malignancies: a multicenter retrospective study

**DOI:** 10.3389/fphar.2026.1912031

**Published:** 2026-09-16

**Authors:** Ranliang Cui, Yue Cao, Yueguo Li

**Affiliations:** 1 Department of Laboratory, Tianjin Medical University Cancer Institute & Hospital, National Clinical Research Center for Cancer, Tianjin Key Laboratory of Digestive Cancer, State Key Laboratory of Druggability Evaluation and Systematic Translational Medicine, Tianjin’s Clinical Research Center for Cancer, Tianjin, China; 2 Department of Laboratory, Tianjin First Central Hospital, School of Medicine, Nankai University, Tianjin, China

**Keywords:** blood biomarkers, blood-based risk stratification, digestive malignancies, GDF15, machine learning

## Abstract

**Background:**

Digestive malignancies remain a major cause of cancer-related mortality worldwide. Current diagnostic approaches are often limited by invasiveness, cost, and accessibility. We aimed to develop a blood-based risk stratification model for identifying individuals at increased risk of digestive malignancies.

**Methods:**

We retrospectively collected clinical data from 667 patients with digestive malignancies and 795 non-cancer controls. A machine learning model was developed by integrating GDF15 with selected routine blood biomarkers. Model performance was evaluated using measures of discrimination, risk reclassification, and SHAP analysis. Blood GDF15 levels were measured by chemiluminescent immunoassay, and tissue expression was assessed by immunohistochemistry.

**Results:**

The GDF15-based machine learning model showed favourable performance in the independent validation cohort (AUROC = 0.886). Compared with conventional tumor markers alone, the addition of GDF15 improved model discrimination (AUROC, 0.856 vs. 0.777; *P* < 0.001) and significantly enhanced risk reclassification (NRI = 0.858; IDI = 0.151). GDF15 also contributed to the identification of patients with negative conventional tumor markers and was among the most influential variables in the model according to SHAP analysis. Higher circulating GDF15 levels were associated with larger tumor size, shorter progression-free survival (HR = 2.68), and increased expression in tumor tissues.

**Conclusion:**

Integrating GDF15 with routine blood biomarkers enables effective risk stratification of individuals at increased risk of digestive malignancies. This blood-based model may provide a practical approach for risk stratification and help identify individuals who may benefit from further diagnostic evaluation.

## Introduction

According to the World Health Organization, by 2050, there will be as many as 11 million cases of cancer in high-income countries, up from about 8.3 million in 2022 (World Health Organization, 2024). Digestive malignancies represent a primary factor in global cancer mortality, and numerous patients receive diagnoses during advanced phases; consequently, the clinical outlook is typically unfavorable (Li et al., 2024). Therefore, the identification and stratification of individuals at high risk of cancer have become increasingly important (Assarzadegan and Montgomery, 2021). Risk stratification approaches that integrate biomarkers with clinical characteristics may facilitate individualized risk assessment and support clinical decision-making (Sun et al., 2024; Giorgi Rossi et al., 2026).

Currently, evidence suggests that risk prediction models based on electronic health records (EHRs) have been developed and applied for risk assessment across various digestive system malignancies, including esophageal, gastric, colorectal, liver, and pancreatic cancers, with encouraging predictive performance (Jung et al., 2024). Among these malignancies, colorectal cancer has been extensively investigated in the context of risk stratification. Emerging approaches have incorporated clinical risk factors, genetic susceptibility, biomarkers, and multivariable prediction models to improve individual risk assessment (Hull et al., 2020). Traditional blood-based biomarkers, including carcinoembryonic antigen (CEA) and carbohydrate antigen 19–9 (CA19-9), also have relatively low sensitivity and specificity and are therefore not suitable for the early detection or risk stratification of cancer (Tang et al., 2022).

GDF15 is a stress-responsive cytokine involved in the development and progression of digestive malignancies. Research has shown that it promotes the growth, spread, and metastasis of tumors in esophageal, gastric, and pancreatic cancer (Okamoto et al., 2020; Xu et al., 2020; Zhao et al., 2020), and elevated levels in the blood are associated with diagnosis, prognosis, and other predictions for hepatocellular carcinoma (Cheng et al., 2020; Myojin et al., 2022). Despite the above results, the position of this method in the combined control of digestive malignancies is still unknown. At the same time, most of the applications of machine learning in digestive cancer have focused on risk prediction for specific types of cancer, such as colorectal cancer, cholangiocarcinoma, and esophageal cancer, and a general model that can simultaneously identify multiple digestive malignancies is currently missing (Yin et al., 2024; Liu et al., 2024; Yu et al., 2025).

To address the above deficiencies in current research, we used clinical data from individuals with and without digestive malignancies to develop and compare various prediction models based on advanced statistical and machine learning techniques (Gangwani et al., 2026; Saunders et al., 2025), and identified GDF15 as a potential key indicator. The primary aim of this study was to develop a risk stratification model for digestive system malignancies to evaluate its potential for identifying individuals at increased risk and facilitate the identification of individuals at higher risk. The model’s discrimination, calibration, and clinical utility were comprehensively evaluated through internal validation and independent test cohort. SHAP analysis was further applied to interpret feature contributions, identify the factors influencing model predictions, and improve model transparency (Alghafees et al., 2024).

## Materials and methods

### Study population and outcome definition

Clinical data were retrospectively collected from two centers, and an exploratory blood-based risk stratification model for digestive malignancies was developed and evaluated in an independent test cohort. The research used the TRIPOD (Transparent Reporting of a multivariable prediction model for Individual Prognosis Or Diagnosis) (Collins et al., 2024) and the Stepwise Guide for the Development of Clinical Predictive Models (Efthimiou et al., 2024). The design of the study is shown in Figure 1.

**FIGURE 1:**
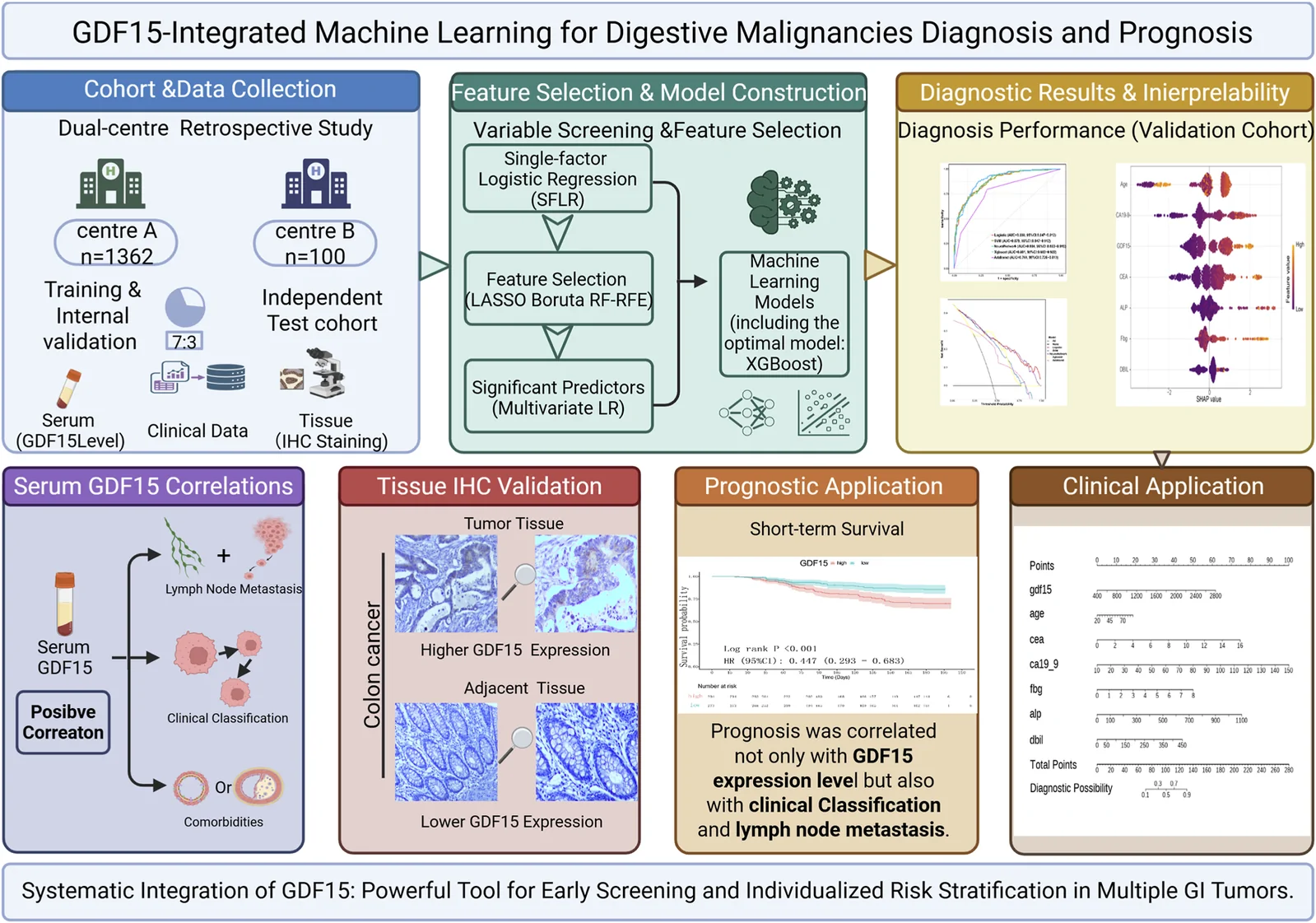
Graphical Abstract. First line: This multicenter retrospective study collected data from 1,462 cancer pre-screening cases, of which 1,362 were from Centre A and were used for model development and internal validation (split in a 7:3 ratio), while 100 were from Centre B and were used for external validation. The study collected serum GDF15 levels, clinical variables, and tumor tissue samples and employed LASSO, Boruta, and RF-RFE algorithms to identify predictive factors; models were constructed using logistic regression and machine learning, and model performance was evaluated using ROC curves, DCA, and SHAP. Second line: Based on the optimal model, a nomogram was built, and further analyses included GDF15-stratified survival, immunohistochemical assessment, and correlations between GDF15 expression and tumor burden. Created with BioRender.com.

Baseline characteristics of the patients, radiological findings, laboratory tests and clinicopathological data were all retrieved from the electronic medical record. Eligible participants were aged ≥40 years and had complete baseline clinical and laboratory data. Patients with digestive malignancies were required to have a confirmed diagnosis based on histopathological or imaging findings. Individuals with a history of malignancy, prior anti-cancer treatment, or severe comorbidities that might significantly affect GDF15 levels (such as advanced heart failure, end-stage renal disease, liver cirrhosis, and severe active infection (Ceelen et al., 2022; Echouffo-Tcheugui et al., 2022; Delrue et al., 2023)) were excluded. The specific diagnostic criteria are shown in the Supplementary Materials. Participants missing important clinical or laboratory data that could not be obtained from medical records were also excluded.

A total of 1,462 participants were included, comprising patients with digestive malignancies and non-cancer controls. Among these, 1,362 participants were collected at Tianjin Medical University Cancer Hospital from April 2024 to February 2026, and 100 participants were collected at Tianjin First Central Hospital from October 2025 to February 2026 (Figure 2, Supplementary Material: Specific criteria for defining comorbidities and Study population). A total of 31 candidate variables, including demographic characteristics, comorbidities, laboratory parameters, and tumor-related features, were collected (Supplementary Material: Collection of variables).

**FIGURE 2:**
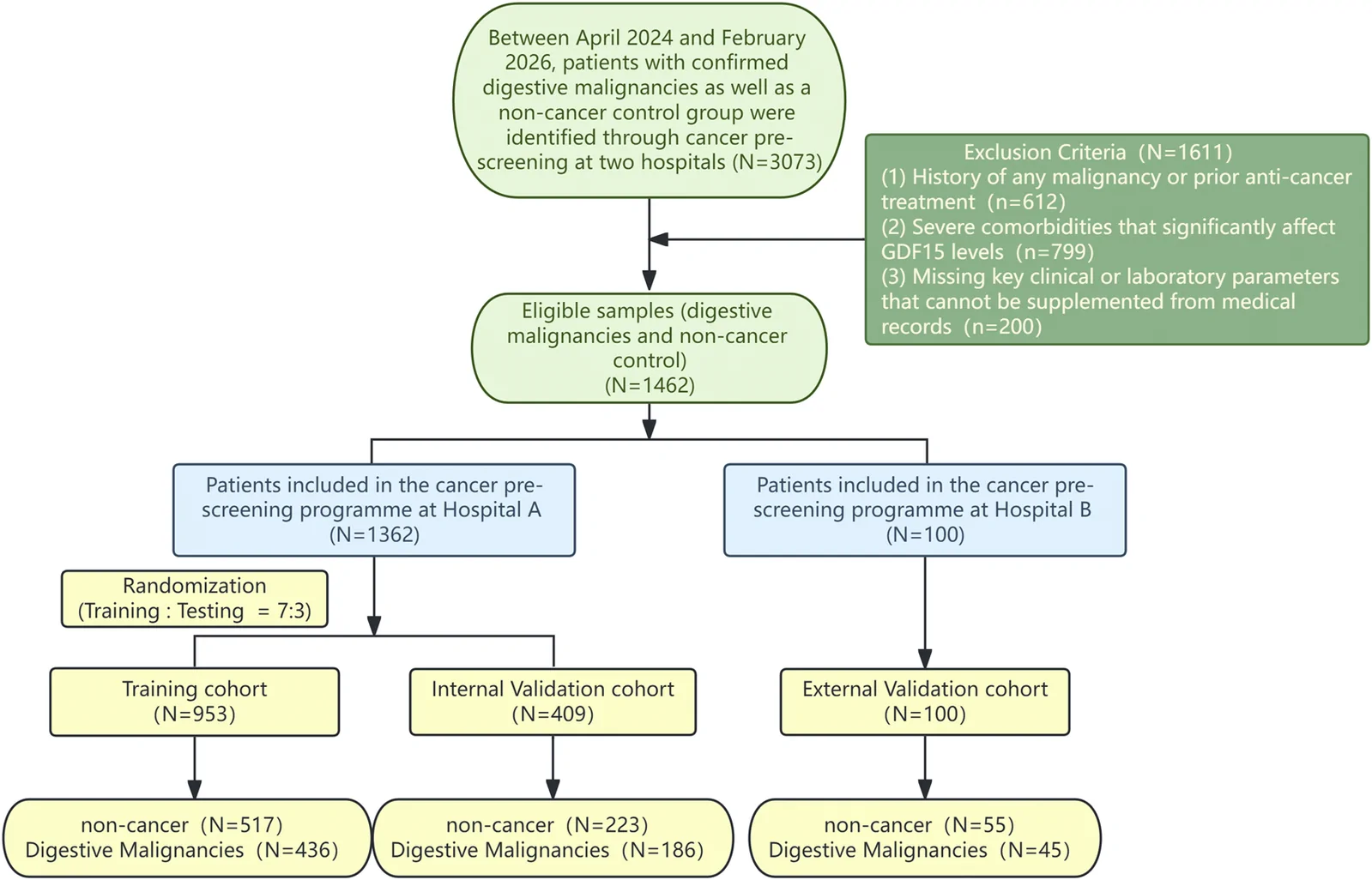
Flowchart of included participants.

The primary outcome was a confirmed diagnosis of digestive malignancy. Subgroup analyses were performed according to tumor stage, lymph node status, distant metastasis status, and other clinicopathological characteristics. Survival information was obtained through telephone follow-up. Progression-free survival (PFS) was defined as the interval from diagnosis to disease progression, metastasis, or death, with follow-up continuing until 1 April 2026 (Supplementary material: Follow-up data).

### Ethical statement

The study was approved by the Ethics Committee of Tianjin Medical University Cancer Institute and Hospital (No. BC20260528) and the Science and Technology Ethics Committee of the First Central Hospital of Tianjin (No. KYAP2026-149). Informed consent was waived owing to the retrospective design and anonymized data collection. The study was registered in the China Clinical Trial Registry (No. ChiCTR2600121976).

### Measurement of GDF15 and tissue validation

Serum GDF15 concentrations were measured using a direct chemiluminescence assay according to the manufacturer’s instructions (Macke Biotechnology Co., Ltd.). All quality-control results met predefined acceptance criteria.

Tumor tissues and matched adjacent normal tissues from 50 patients with digestive malignancies were collected at Tianjin Medical University Cancer Institute and Hospital (Supplementary material: Tissue sample collection). GDF15 expression was assessed by immunohistochemistry (IHC) using a GDF15 antibody (Wuhan Sanying Biotechnology Co., Ltd., China; catalogue number: 27455-1-AP; dilution 1:200) and independently scored by two blinded pathologists using the H-score system (0–300). Differences in GDF15 expression between paired tissues were analyzed using the Wilcoxon signed-rank test. Detailed staining procedures and scoring criteria are provided in the Supplementary Methods.

### Model development and validation

Continuous variables were analyzed as raw values, and gender was coded as female = 0 and male = 1. Variables with >20% missing values were excluded, and remaining missing data were imputed using the MICE package in R (Supplementary Figure S1; Supplementary Tables S1, S2) (Kwak and Kim, 2017). Sample size requirements were estimated using the pmsampsize package in R (version 4.4.2). Based on an expected c-statistic of 0.85, Cox–Snell R^2^ of 0.20, 23 candidate predictors, and an event rate of 45%, the minimum required sample size was estimated to be 519 participants. The final sample of 1,462 participants exceeded this threshold (Riley et al., 2020).

The Tianjin Medical University Cancer Institute and Hospital dataset (n = 1,362) was randomly divided into training (70%) and internal validation (30%) cohorts using stratified sampling. The Tianjin First Central Hospital dataset (n = 100) served as an independent test cohort. Candidate variables were screened by univariable logistic regression and further selected using least absolute shrinkage and selection operator (LASSO), Boruta feature selection, and random forest–recursive feature elimination (RF-RFE) with 10-fold cross-validation. Variables consistently retained across methods were entered into multivariable logistic regression to identify independent predictors (Supplementary Table S3).

Five machine-learning algorithms (logistic regression, support vector machine [SVM], neural network [NN], extreme gradient boosting [XGBoost], and adaptive boosting [AdaBoost]) were developed using three repetitions of 10-fold cross-validation. Hyperparameter settings and optimization results are provided in Supplementary Tables S4, S5.

Model performance included discrimination (AUC with 95% CI), calibration (Brier score and Hosmer-Lemeshow test (Angraal et al., 2020)), and clinical utility (DCA). Youden’s index was used to select an appropriate cut-off value; sensitivity, specificity, accuracy, PPV, NPV, and F1 score were computed. Internal robustness assessment was performed using repeated cross-validation and bootstrap resampling (Davies et al., 2022). Differences in area under the receiver operating characteristic curve (AUC) were evaluated using the DeLong test, and net reclassification improvement (NRI) and integrated discrimination improvement (IDI) were used to assess risk reclassification (Angraal et al., 2020). Model interpretability was assessed using SHapley Additive exPlanations (SHAP). The detailed SHAP analysis workflow is described in the Supplementary Methods.

### Statistical analysis

Statistical analyses were conducted using R (version 4.4.2), RStudio (2024.12.0-467), and SPSS (version 27.0). Continuous variables were compared using Student's t-test or the Wilcoxon rank-sum test, as appropriate, whereas categorical variables were compared using the chi-square test or Fisher’s exact test, as appropriate. All statistical tests were two-sided, and *P* < 0.05 was considered statistically significant. Kaplan–Meier analysis and Cox proportional hazards models were used to assess PFS, with hazard ratios (HRs) reported together with 95% confidence intervals. Follow-up duration was estimated using the reverse Kaplan–Meier method. Details of machine-learning packages are provided in Supplementary Table S6.

## Results

### Baseline characteristics

A total of 1,462 participants were divided into training (n = 953), internal validation (n = 409), and independent test (n = 100) cohorts. The median age of the study population was 52.00 years (Supplementary Table S7). Compared with non-cancer controls, patients with digestive malignancies exhibited significant differences in several laboratory parameters, most notably CEA, CA19-9, and GDF15 (*P* < 0.05) (Table 1).

**TABLE 1:** Baseline patient characteristics in the training and validation cohort.

Variables	Training cohort (N = 953)			Internal validation cohort (N = 409)			Independent test cohort (N = 100)		
​	Non-cancer (N = 517)	Cancer (N = 436)	*p* value	Non-cancer (N = 223)	Cancer (N = 186)	*p* value	Non-cancer (N = 55)	Cancer (N = 45)	*p* value
Gender	​	​	0.379	​	​	0.284	​	​	0.614
Male	301 (58.2%)	267 (61.2%)	​	119 (53.3%)	110 (59.1%)	​	32 (58.2%)	23 (51.1%)	​
Female	216 (41.7%)	169 (38.7%)	​	104 (46.6%)	76 (40.8%)	​	23 (41.8%)	22 (48.9%)	​
Age	47.0 [38.0; 63.0]	54.5 [46.0; 61.0]	<0.001	48.4 [35.0; 62.0]	51.0 [44.0; 59.0]	0.107	49.7 (12.5)	52.9 (13.1)	0.222
BMI	21.9 [20.6; 23.1]	21.70 [19.8; 23.7]	0.757	21.8 [20.7; 22.8]	21.6 [20.1; 23.8]	0.757	21.8 (2.5)	21.8 (2.3)	0.861
GDF15 (pg/mL)	833.1 [643.6; 1,089.1]	1,216.8 [882.7; 1,560.7]	<0.001	790.2 [630.6; 984.4]	1,193.8 [858.0; 1,596.9]	<0.001	908.2 [743.5; 1,067.9]	1,520.8 [1,385.9; 1,644.3]	<0.001
CEA (ng/mL)	3.0 [2.1; 4.5]	4.7 [3.3; 6.5]	<0.001	3.2 [2.2; 4.3]	4.7 [3.3; 6.1]	<0.001	3.3 [2.8; 3.9]	6.8 [5.7; 7.4]	<0.001
CA19-9 (U/mL)	30.7 [23.0; 41.8]	49.6 [35.3; 67.5]	<0.001	31.6 [23.4; 43.6]	47.0 [33.9; 63.2]	<0.001	22.2 [6.0; 112.4]	78.1 [35.3; 238.6]	0.001
HB (g/L)	129.25 [122.0; 136.7]	130.5 [122.2; 137.5]	0.826	128.1 [120.9; 134.4]	129.6 [121.5; 137.2]	0.53	129.7 (7.3)	129.8 (7.7)	0.925
PLT (×10^9^/L)	219.0 [173.0; 268.0]	230.0 [175.7; 304.25]	0.016	225.0 [174.5; 264.0]	224.0 [168.5; 297.7]	0.522	236.6 (78.0)	216.0 (67.2)	0.16
PT (s)	12.5 [10.1; 15.5]	12.6 [10.3; 15.7]	0.458	12.6 [9.9; 16.1]	12.4 [9.8; 15.4]	0.82	13.6 (4.1)	13.5 (3.5)	0.972
Fbg (g/L)	3.0 [2.3; 3.5]	3.4 [2.7; 4.2]	<0.001	3.1 [2.5; 3.7]	3.3 [2.6; 4.1]	0.002	3.9 (0.6)	3.6 (0.7)	0.045
D-Dimer (ng/L)	444.3 [247.3; 686.2]	461.2 [261.8; 692.3]	0.932	481.2 [273.4; 719.6]	467.1 [283.4; 685.8]	0.936	88.44 [58.96; 117.92]	612.32 [408.12; 816.23]	0.835
ALT (U/L)	37.78 [24.0; 52.2]	37.9 [23.6; 53.3]	0.578	33.8 [23.6; 53.6]	37.1 [25.1; 53.9]	0.434	42.6 [25.5; 58.8]	32.0 [26.6; 42.5]	0.19
AST (U/L)	25.7 [20.2; 31.5]	23.1 [20.1; 28.3]	0.022	24.8 [19.1; 31.2]	23.9 [20.7; 29.2]	0.314	27.3 (8.3)	27.3 (6.8)	0.973
ALP (U/L)	84.4 [38.6; 131.1]	125.5 [53.1; 204.5]	<0.001	76.2 [32.7; 136.2]	133.2 [66.1; 218.1]	<0.001	109.5 [61.7; 148.1]	135.1 [66.8; 194.3]	0.097
GGT (U/L)	32.4 [14.6; 49.4]	33.9 [16.7; 52.3]	0.419	34.4 [17.9; 53.5]	28.5 [13.3; 44.1]	0.029	35.8 [19.8; 49.8]	43.7 [29.6; 58.1]	0.033
TP (g/L)	70.5 [66.9; 74.8]	71.3 [66.9; 74.8]	0.388	70.4 [66.9; 73.8]	71.3 [66.9; 74.9]	0.707	71.2 (4.1)	72.1 (4.4)	0.362
ALB (g/L)	42.8 (5.4)	44.69 (5.6)	<0.001	42.7 (5.8)	44.7 (5.45)	<0.001	44.4 (6.3)	42.3 (5.8)	0.089
DBIL (mol/L)	4.2 [3.0; 6.0]	5.5 [4.1; 9.5]	<0.001	4.0 [3.0; 5.9]	5.9 [3.7; 12.8]	<0.001	18.7 [9.0; 34.1]	13.8 [4.7; 28.1]	0.204
UREA (mmol/L)	5.5 [3.40; 7.98]	5.3 [3.5; 8.3]	0.651	5.7 [3.9; 7.8]	5.1 [3.2; 7.6]	0.062	5.5 (2.6)	5.6 (2.9)	0.867
eGFR (mL/min/1.73 m^2^)	87.82 [64.97; 109.10]	84.65 [64.10; 104.28]	0.093	88.22 [58.81; 107.37]	82.67 [60.63; 104.23]	0.335	86.22 [56.81; 105.24]	83.67 [59.23; 103.25]	0.135
BNP (pg/mL)	12.57 [10.00; 23.00]	12.65 [10.00; 26.23]	0.584	12.57 [10.00; 22.28]	11.01 [10.00; 23.62]	0.477	11.57 [9.00; 21.08]	12.01 [11.00; 24.52]	0.377
CRP (mg/L)	16.00 [9.00; 22.00]	9.11 [2.25; 20.59]	<0.001	15.00 [7.00; 23.00]	7.81 [2.61; 16.96]	<0.001	14.00 [8.00; 21.00]	8.21 [1.61; 15.06]	<0.001
GLU (mmol/L)	6.10 [5.30; 6.80]	6.20 [5.40; 6.80]	0.415	6.00 [5.15; 6.70]	5.90 [5.30; 6.70]	0.988	6.50 [5.75; 6.90]	5.80 4.30; 6.90]	0.778
Comorbidities	​	​	0.339	​	​	0.5	​	​	0.43
No	459 (91.62%)	405 (89.60%)	​	217 (90.79%)	150 (88.24%)	​	50 (90.10%)	41 (91.11%)	​
Yes	42 (8.38%)	47 (10.40%)	​	22 (9.21%)	20 (11.76%)	​	5 (9.90%)	4 (8.89%)	​

GDF-15, growthdifferentiationfactor-15; BMI, bodymassindex; eGFR, estimated glomerular filtration rate.

### Model development and validation

No significant multicollinearity or correlation issues were detected. Candidate variables were screened using univariable logistic regression and multiple feature-selection approaches. Seven independent predictors were ultimately identified: GDF15, CEA, CA19-9, age, Fbg, ALP, and DBIL (Supplementary Figure S2; Supplementary Tables S8, S9). Five machine-learning algorithms were subsequently developed and compared. Among them, XGBoost consistently achieved the best performance and was selected as the final model. The AUROCs of the XGBoost model were 0.920 (95% CI: 0.904–0.937) in the training cohort, 0.891 (95% CI: 0.860–0.922) in the internal validation cohort, and 0.886 (95% CI: 0.822–0.949) in the independent test cohort (Figures 3A,C,E). Furthermore, the XGBoost model has demonstrated favourable discriminatory ability across different types of digestive malignancies (Supplementary Table S10).

**FIGURE 3:**
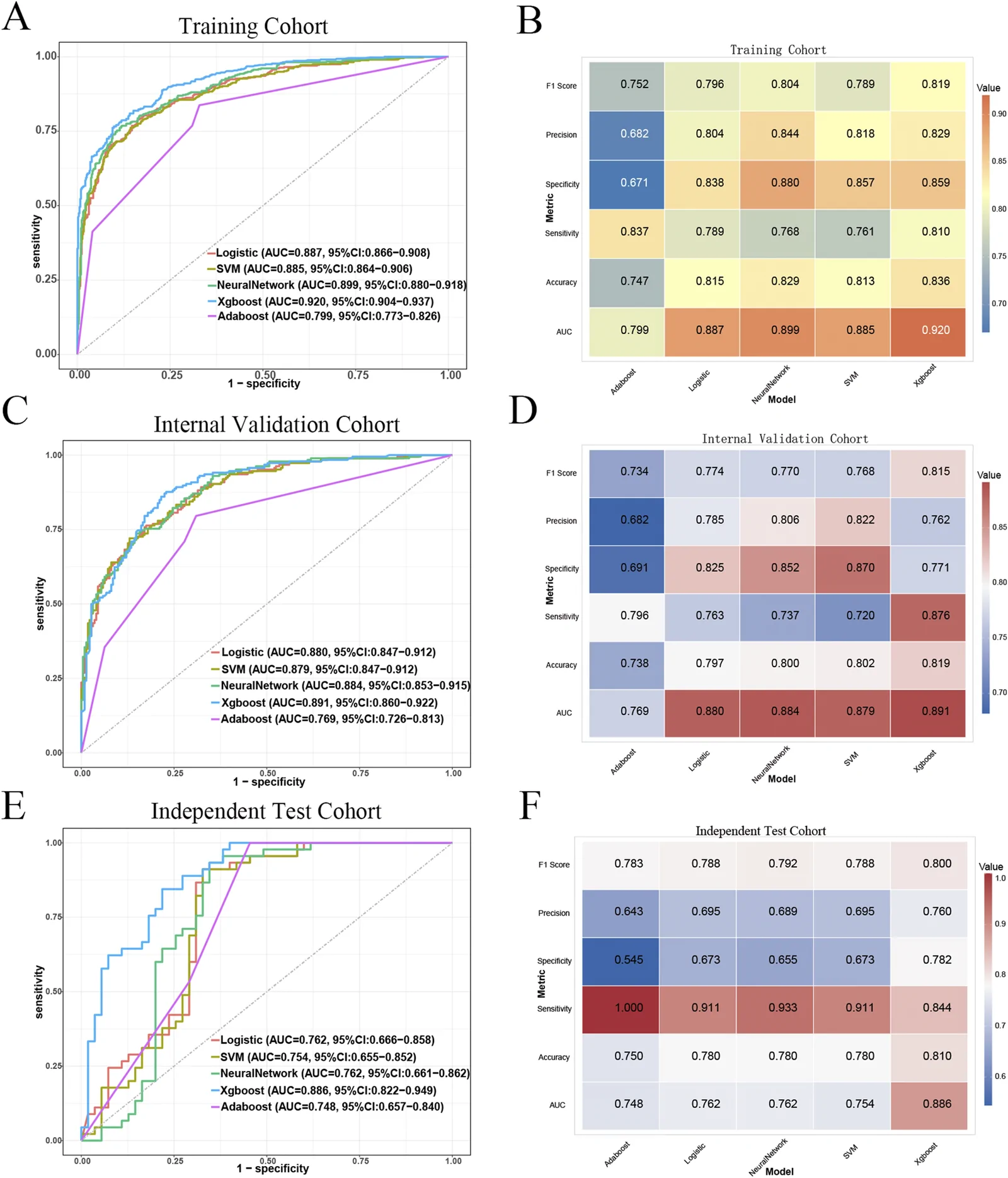
Performance and Evaluation of Five Models: **(A,C,E)** ROC curves in the training cohort, internal validation cohort, and independent test cohort, respectively. **(B,D,F)** The heatmap visually summarizes the comprehensive performance of the five models across six core metrics (AUC, accuracy, sensitivity, specificity, precision, and F1 score) in the training, internal validation, and independent test cohorts, respectively.

Calibration analysis showed acceptable agreement between predicted and observed risks in the training and internal validation cohorts, with non-significant Hosmer–Lemeshow test results and Brier scores ranging from 0.10 to 0.25 (Supplementary Table S11). Decision curve analysis indicated a consistent net clinical benefit across threshold probabilities from 0% to 75% (Supplementary Figure S3). Detailed performance metrics are provided in Figures 3B,D,F and Supplementary Table S12.

Using the optimal cutoff determined by the Youden index, XGBoost achieved the highest overall diagnostic performance and F1 score in the validation cohort (Supplementary Figure S4). The robustness of the model was further confirmed through 10-fold cross-validation and 100 bootstrap resamples, which demonstrated stable discrimination, calibration, and clinical utility (Supplementary Figure S5).

### Added value of GDF15 and model interpretation

To quantify the contribution of GDF15, the XGBoost model (Model A) was compared with conventional tumor marker-based models. Model A showed better discrimination than the traditional tumor marker model (CA19-9 + CEA) (Model C; AUC = 0.822; *P* < 0.001). Inclusion of GDF15 in the traditional model (Model B) increased the AUC to 0.861, and it was still significantly higher than that of Model C (*P* < 0.001). Model A further improved the discrimination, calibration and clinical benefits compared with both comparator models (Figure 4; Table 2; Supplementary Figure S6). The model demonstrated acceptable discriminatory performance across different digestive malignancy subgroups, with AUROC values ranging from 0.838 to 0.907 (Supplementary Table S13).

**FIGURE 4:**
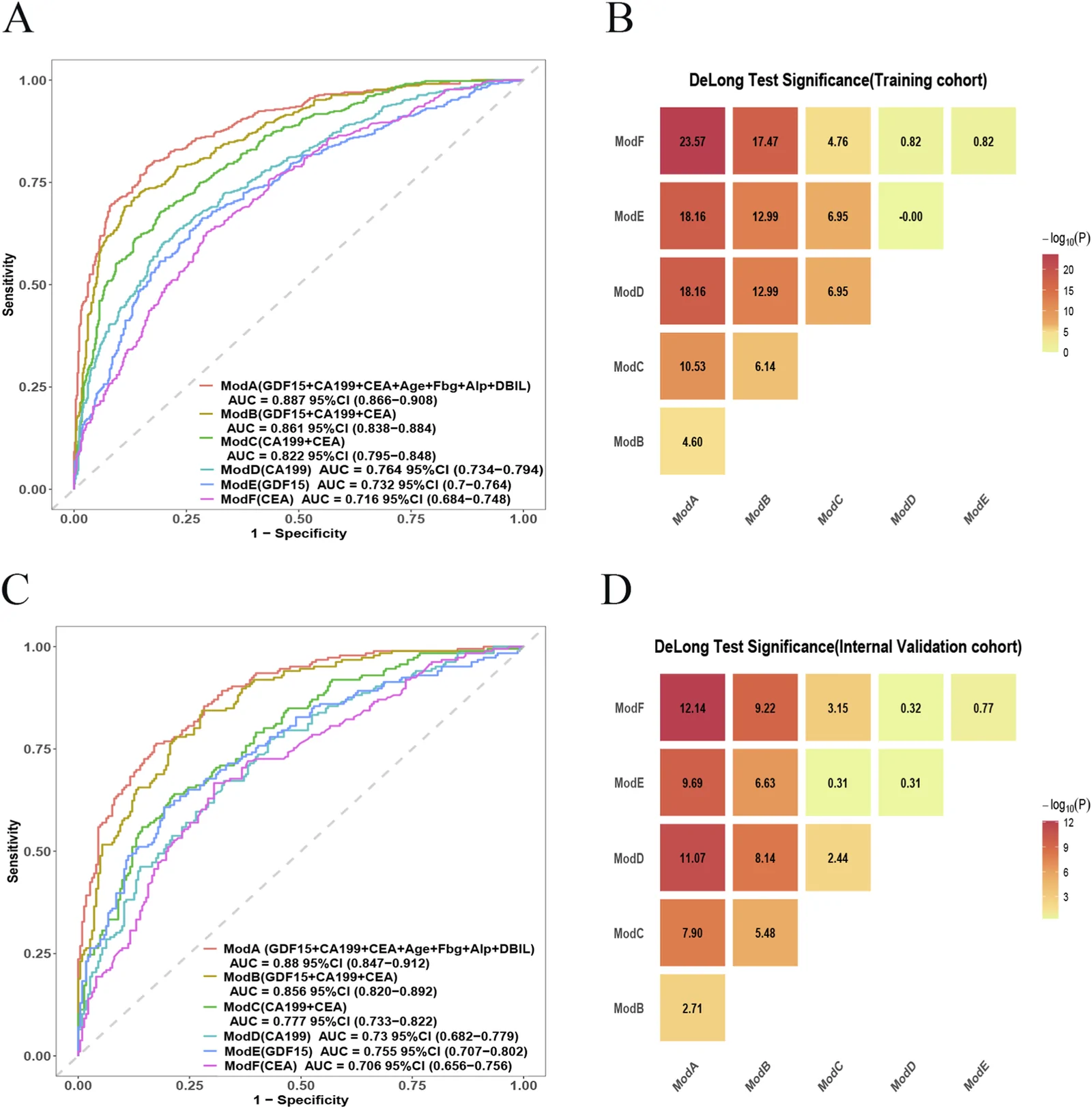
Assessment of the discriminatory ability of different predictive models in the training and internal validation cohorts. **(A,B)** ROC curves for individual indicators and composite models in the two cohorts. **(C,D)** AUC significance heatmaps calculated using the DeLong method. Color represents –log10(P); a higher intensity of blue indicates a larger –log10(P) value, corresponding to a smaller P-value and greater statistical significance. Note: Model Definitions: Model A: GDF15 + CA19-9 + CEA + Age + Fbg + ALP + DBIL. Model B: GDF15 + CA19-9 + CEA. Model C: CA19-9 + CEA. Model D: CA19-9. Model E: GDF15. Model F: CEA.

**TABLE 2:** Comparison of the overall discriminatory power of different models on the training and validation cohort.

Model	AUC [95% CI]	Z	*p*-value	NRI [95% CI]	*p*-value	IDI [95% CI]	*p*-value
Training cohort							
Model A	0.887 [0.866–0.908]	​	​	​	​	​	​
Model B	0.861 [0.838–0.884]	4.2145	<0.001	0.547 [0.425–0.669]	<0.001	0.065 [0.048–0.081]	<0.001
Model C	0.822 [0.795–0.848]	4.9555	<0.001	0.700 [0.581–0.819]	<0.001	0.089 [0.070–0.1073]	<0.001
Model D	0.764 [0.734–0.794]	5.3044	<0.001	0.611 [0.490–0.731]	<0.001	0.096 [0.077–0.115]	<0.001
Model E	0.732 [0.700–0.764]	1.4357	0.151	0.177 [0.050–0.304]	<0.05	0.046 [0.011–0.08]	<0.05
Model F	0.716 [0.684–0.748]	0.6794	0.496	0.118 [−0.008–0.246]	0.067	0.027 [−0.00–0.059]	0.097
Validation cohort							
Model A	0.88 [0.847–0.912]	​	​	​	​	​	​
Model B	0.856 [0.820–0.892]	2.3366	<0.05	0.4403 [0.253–0.627]	<0.001	0.069 [0.0438–0.0954]	<0.001
Model C	0.777 [0.733–0.822]	5.3999	<0.001	0.858 [0.683–1.032]	<0.001	0.151 [0.1154–0.1876]	<0.001
Model D	0.730 [0.682–0.779]	2.9243	<0.05	0.596 [0.411–0.780]	<0.001	0.073 [0.0478–0.0993]	<0.001
Model E	0.755 [0.707–0.802]	−0.6863	0.492	−0.198 [−0.391 to −0.004]	<0.05	−0.046 [−0.101–0.010]	0.106
Model F	0.706 [0.656–0.756]	1.3723	0.17	0.2519 [0.058–0.445]	<0.05	0.086 [0.034–0.137]	<0.05

Model A: GDF15 + CA19-9 + CEA + Age + Fbg + ALP + DBIL, Model B: GDF15 + CA19-9 + CEA, Model C: CA19-9 + CEA, Model D: CA19-9, Model E: GDF15 and Model F: CEA; NRI, Net Reclassification Improvement; IDI, Integrated Discrimination Improvement. B vs. A, C vs. B, D vs. C, E vs. D, F vs. E.

Reclassification analysis showed that adding GDF15 significantly improved risk classification (Model B vs. Model C: NRI = 0.858), and the full XGBoost model also provided some improvement (Model A vs. Model B: NRI = 0.4403, IDI = 0.069; *P* < 0.001. SHAP analysis found that CA19-9, CEA and GDF15 were the three main reasons for model prediction changes. GDF15 consistently ranked among the top variables, and was associated with a higher probability of digestive malignancy in the model (Figure 5; Supplementary Figure S7).

**FIGURE 5:**
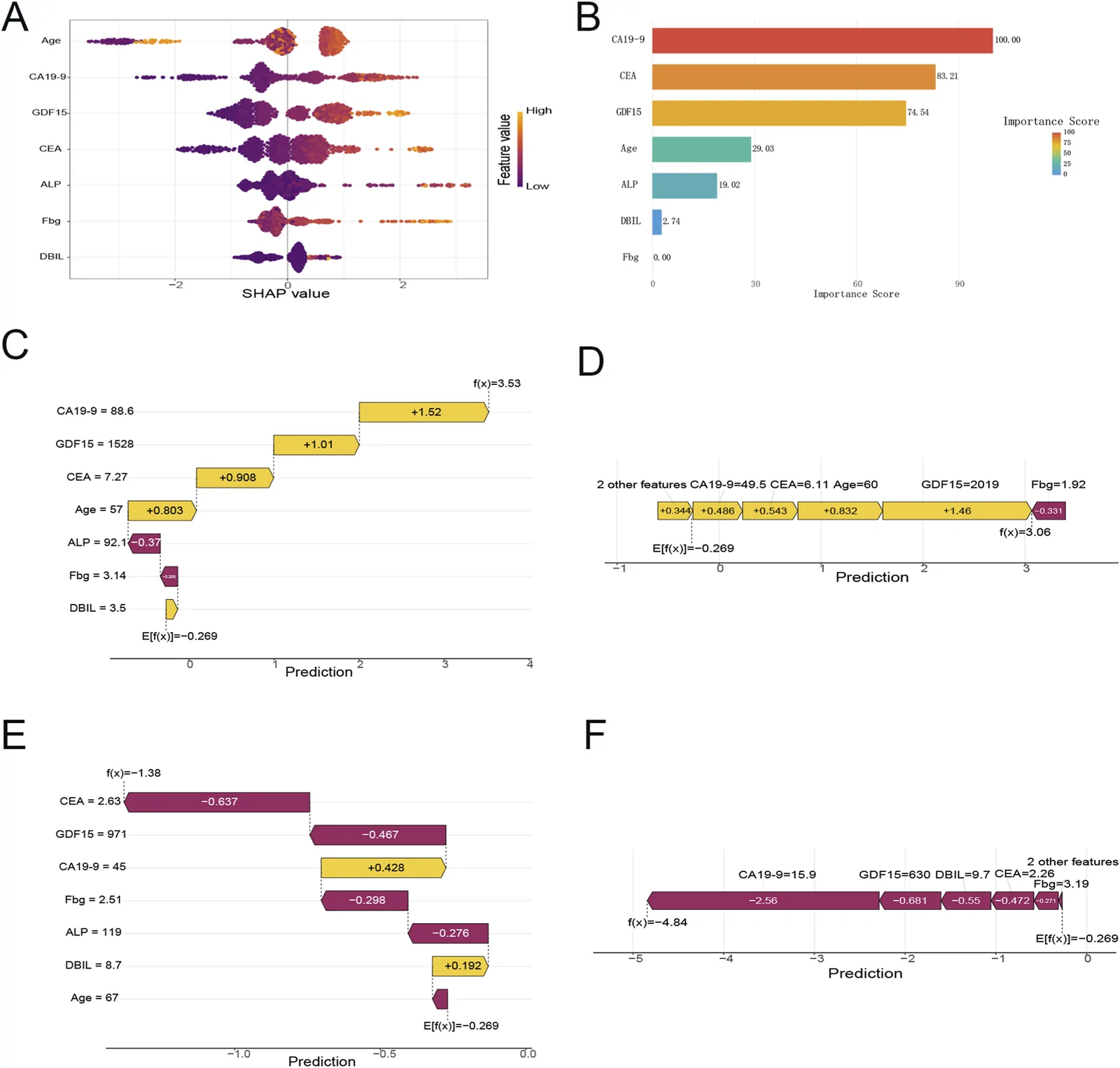
SHAP analysis. **(A)** SHAP summary plot of the XGBoost model. **(B)** Feature importance bar plot of the XGBoost model. **(C,D)** SHAP feature contribution plots for high-risk samples in the digestive malignancies. **(E,F)** SHAP feature contribution plots for low-risk samples in the non-cancer controls.

### Additional clinical analyses and prognostic relevance

Subgroup analysis shows that discrimination ability for disease stage, risk stratification and lymph node metastasis status is moderate, with AUROC values ranging from 0.75 to 0.77 (Supplementary Figure S8). Performance was relatively poor for the distant metastasis model. Among people negative for the traditional tumor markers, GDF15 had a favorable diagnostic performance with AUROCs of 0.73–0.78. Therefore, suggesting potential value for risk stratification among individuals with negative conventional tumor markers (Supplementary Figure S9).

Analysis by age group showed different optimal cut-off values for GDF15 for age groups (AUROC = 0.598). A multi-variable model including age, estimated glomerular filtration rate (eGFR), and GDF15 did not show a significant association with the risk of complications (*P* > 0.05) (Supplementary Figure S10).

A nomogram based on the seven-variable model for individualized risk assessment has been developed. Risk stratifications generated by XGBoost effectively partitioned subjects into low-, medium-, and high-risk categories within the respective training and validation cohorts and accurately reflected the empirical prevalence of digestive malignancies. GDF15 contributed substantially to the overall risk estimation and was identified as one of the key variables supporting individualized risk stratification (Supplementary Figure S11).

The median follow-up time was 216 days (95% CI: 201–262 days). Kaplan-Meier analysis shows that PFS is associated with the expression level of GDF15 and several other factors, such as clinical stage, lymph node metastasis, distant metastasis, and overall metastatic status (*P* < 0.05), but gender is not associated with this outcome (*P* > 0.05) (Supplementary Figure S12). Multivariable Cox regression analysis also identified GDF15, clinical subgroup, and distant metastasis as independent predictors of PFS (Supplementary Tables S14, S15).

### Tissue validation of GDF15 expression

Immunohistochemical analysis showed a significantly higher GDF15 expression in the tissues of digestive malignancy compared with those of adjacent normal tissues. In line with the above results, the concentration of serum GDF15 positively correlated with the tissue GDF15 H-score (r = 0.31, *P* = 0.0014) (Figure 6; Supplementary Figure S13).

**FIGURE 6:**
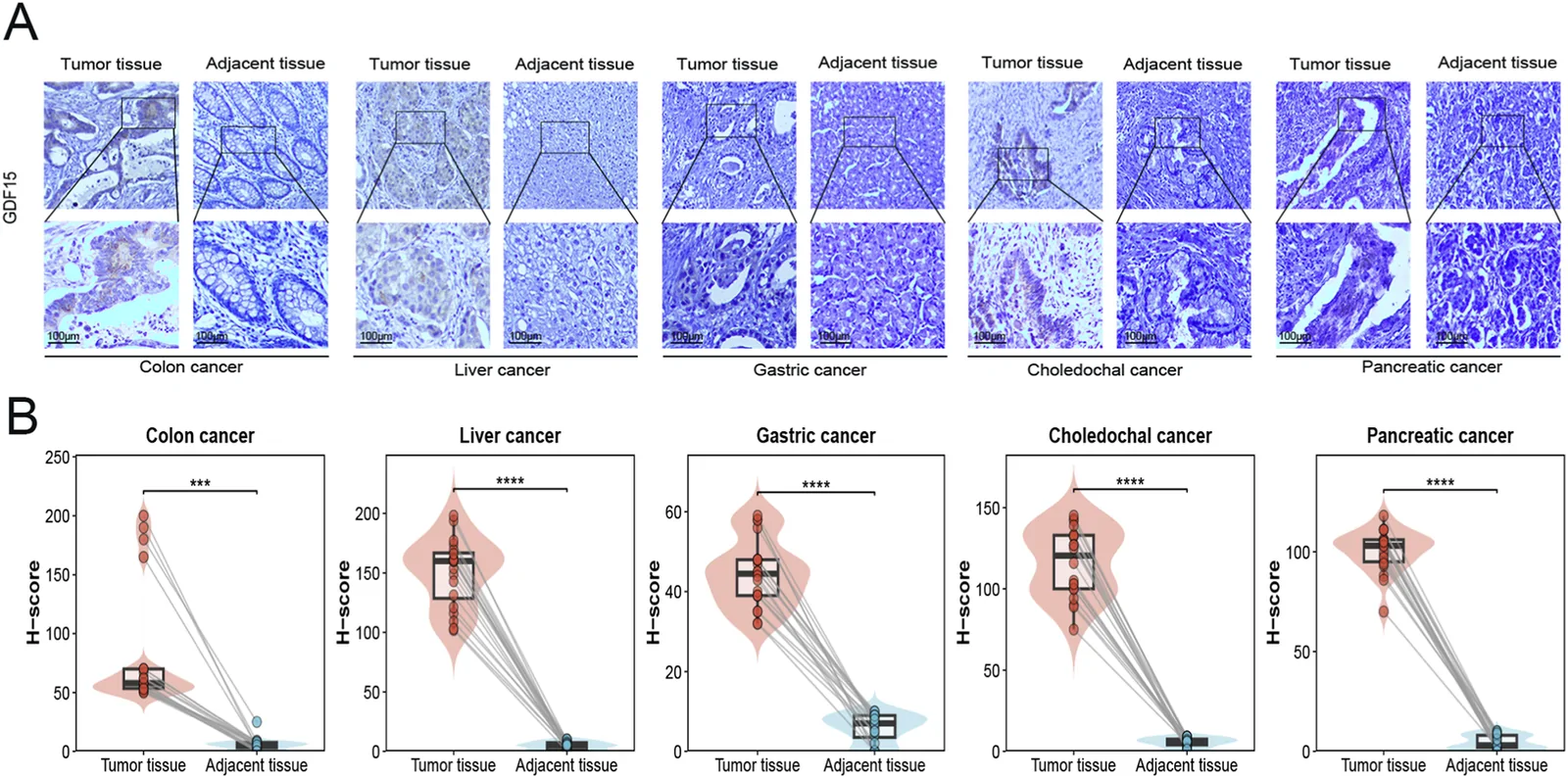
GDF15 expression in digestive malignancies by IHC. **(A)** IHC staining for GDF15 in five types of digestive malignancies and their corresponding adjacent tissues (×200). **(B)** Quantitative comparison of GDF15 H-score in digestive malignancies and adjacent tissues. Statistical significance was defined as ***P < 0.001 and ***P < 0.0001.

## Discussion

In this two-center retrospective study, we developed and independently validated a blood-based risk stratification model for digestive malignancies using GDF15 and easily obtained clinical data. The model showed consistent performance across independent cohorts and demonstrated potential utility for stratifying individuals according to cancer risk. Incorporation of GDF15 improved discrimination, risk reclassification, and clinical utility over traditional marker-based approaches, supporting its potential value as a complementary biomarker for cancer risk assessment.

The composition of the final model is biologically plausible. In addition to GDF15, other traditional tumor markers (CEA and CA19-9), age, and laboratory data related to blood clotting, liver and bile duct conditions, and other functions were also included in the model. These variables are likely to be different parts of host-tumor interaction, and together they provide a complete view of cancer-associated biological changes. Suggesting that GDF15 may capture additional biological information not fully reflected by traditional tumor markers or routine laboratory measurements.

The additional contribution of GDF15 compared with conventional tumor markers may be related to its ability to reflect broader tumor-associated biological alterations, whereas CEA and CA19-9 mainly reflect tumor burden and secretory activity (Fichtner et al., 2024; Zhou et al., 2020). It is worth noting that GDF15 may provide complementary information among individuals with negative traditional tumor marker results, suggesting its potential value for improving risk stratification when combined with conventional biomarkers. Notably, GDF15 provided additional discriminatory information among individuals with negative conventional tumor markers, suggesting its potential value as a complementary biomarker for risk stratification rather than a standalone diagnostic marker.

Previous studies have demonstrated that advancing age is associated with an increased risk of cancer development (Siegel et al., 2023). This finding is consistent with the inclusion of age as an important predictive variable in our digestive malignancy risk model. In line with previous reports, higher GDF15 levels are associated with a greater tumor burden, more advanced disease staging, and more pronounced lymph node involvement (Fichtner et al., 2024; Sawant and Borthakur, 2025; Lee et al., 2025). Adjusting for known clinical and pathological factors, GDF15 was still independently associated with poor prognosis. Therefore, GDF15 may serve as a complementary biomarker for risk assessment and prognostic evaluation in digestive malignancies.

The primary value of this model lies not in replacing established diagnostic procedures, but in providing an exploratory blood-based risk stratification approach. By integrating GDF15 with conventional clinical variables and biomarkers, the model may help identify individuals with different levels of cancer risk and support personalized decisions regarding further clinical evaluation.

Several limitations should be acknowledged. Firstly, the retrospective study design may have selection bias and residual confounding factors. Second, although an independent test cohort was included, its relatively small sample size and imbalanced distribution of digestive malignancy subtypes may have limited the robustness of model evaluation. Subgroup analyses demonstrated acceptable discrimination across different malignancy categories; however, variations in model performance may be influenced by differences in sample size, clinical characteristics, and underlying biological heterogeneity among malignancy categories, particularly hepatobiliary and pancreatic malignancies. Third, the non-cancer control cohort primarily consisted of individuals without digestive malignancies who met strict exclusion criteria for major comorbidities. Although this approach helped reduce potential confounding during model development, it may have introduced selection bias by resulting in a relatively homogeneous control population compared with real-world clinical settings. Such a limited representation of clinical heterogeneity may lead to an overestimation of model discrimination and may affect its generalizability when applied to populations with benign digestive diseases or other complex comorbid conditions. Fourth, although the XGBoost model showed promising discrimination performance across the evaluated cohorts, the sample size of individual cancer subgroups was relatively small, particularly in the independent test cohort. Therefore, further validation in larger, well-characterized independent cohorts with adequate representation of different digestive malignancy subtypes is required to confirm the robustness and generalizability of the model. Although GDF15 provided incremental value in the multivariable model, its biological specificity remains an important limitation. As a stress-responsive cytokine, GDF15 can be elevated in various non-malignant conditions and therefore should not be regarded as a tumor-specific biomarker. Given its broad biological response profile, GDF15 is more likely to serve as a complementary biomarker rather than an independent diagnostic indicator. Previous studies in pancreatic and gastric cancers have suggested that integrating GDF15 with conventional tumor-associated biomarkers can improve discriminatory performance, further supporting the potential value of multimarker strategies for enhancing cancer risk stratification (Lee et al., 2025; Liu et al., 2019).

Future research should establish larger multicenter prospective cohorts with broader patient populations and longer follow-up periods to further validate the prognostic value of GDF15 and better define its clinical implications. Incorporating patients with benign digestive diseases and integrating additional molecular markers and imaging features may further improve the predictive performance and clinical utility of GDF15-based risk stratification models for digestive malignancies.

## Conclusion

This study developed and validated a blood-based risk stratification model integrating GDF15 with routine clinical variables for digestive malignancies. The model showed promising performance in risk discrimination, and GDF15 provided incremental information beyond traditional tumor markers. These findings indicate that GDF15 may serve as a complementary biomarker for risk assessment and may assist in identifying individuals who require further diagnostic evaluation.

## Data Availability

The original contributions presented in the study are included in the article/Supplementary Material, further inquiries can be directed to the corresponding author.
