# Supplementary material for: GDF15-integrated blood biomarker panel for risk stratification of digestive malignancies: a multicenter retrospective study
Source: Front Pharmacol. 2026 Sep 16;17:1912031. doi: 10.3389/fphar.2026.1912031 (PMC13623688; doi:10.3389/fphar.2026.1912031)
Supplement: Supplementary file 1 [file Table1.docx]

GDF15-integrated blood biomarker panel for risk stratification of digestive malignancies: a multicenter retrospective study

Ranliang Cui ^1#^,Yue Cao^2#^ ,Yueguo Li ^1*^

[sMaterials	3](#_Toc3498)

[Specific criteria for defining comorbidities	3](#_Toc5059)

[Study population	3](#_Toc32335)

[Collection of variables	3](#_Toc31611)

[Follow-up data	4](#_Toc6170)

[Tissue sample collection	5](#_Toc13575)

[sMethods	5](#_Toc14419)

[Immunohistochemical analysis	5](#_Toc18663)

[H-score method for immunohistochemistry	6](#_Toc3506)

[SHAP analysis workflow	6](#_Toc18926)

[Table S1. Summary of missing values in the stratification variables prior to interpolation	8](#_Toc27342)

[Table S2.Summary of MICE multiple imputation settings	8](#_Toc26402)

[Table S3.characteristics of different feature selection methods	9](#_Toc30268)

[Table S4.Details of the parameters for different machine learning models in the training cohort	9](#_Toc6623)

[Table S5.Hyperparameter tuning strategy and final selected parameters for the XGBoost model	10](#_Toc25751)

[Table S6.Detailed information on the main machine learning software packages	10](#_Toc10809)

[Table S7.Analysis of characteristics in patients with digestive malignancies and non-cancer control group	11](#_Toc21476)

[Table S8.Univariate and multivariate regression analyses of clinical predictive factors	12](#_Toc25438)

[Table S9.All regression coefficients	12](#_Toc23276)

[Table S10.AUC values for different cancer types in the XGBoost model on the training and validation cohorts.	13](#_Toc24576)

[Table S11.The Hosmer-Lemeshaux test for assessing calibration	13](#_Toc13436)

[Table S12.Performance metrics of five machine learning models on the training and validation cohort	14](#_Toc22287)

[Table S13.Subgroup performance of the seven-variable model](#_Toc28086)	15

[Table S14.Multicollinearity analysis of variables included in Cox regression	15](#_Toc21324)

[Table S15.Univariate and multivariate Cox regression	15](#_Toc30678)

[Figure S1.Overview of missing data distribution for included clinical variables	17](#_Toc21635)

[Figure S2. Methods for selecting variables based on different criteria	18](#_Toc16654)

[Figure S3.Performance and Evaluation of Five Models.	19](#_Toc3939)

[Figure S4. Confusion matrices for five machine learning models in the internal validation and independent test cohort	20](#_Toc21873)

[Figure S5.10-fold cross-validation and 100 bootstraps	21](#_Toc26962)

[Figure S6.Evaluation of clinical net benefit, and calibration of different prediction models (training cohort vs. internal validation cohort)	23](#_Toc30641)

[Figure S7.SHAP Summary Analysis and Model Performance Evaluation	24](#_Toc25944)

[Figure S8.Evaluation of the model’s predictive performance across different clinical progression strata	25](#_Toc18026)

[Figure S9.AnalysisS6. Analysis of ROC curves for tumor markers in corresponding negative populations	26](#_Toc23696)

[Figure S10.Age Correlation and Stratified Predictive Performance of GDF15](#_Toc1981)	[28](#_Toc12360)

[Figure S11.Risk stratification and prediction chart for malnutrition in digestive malignancies	28](#_Toc8102)

[Figure S12. Association of Clinical Characteristics with Patient Survival Prognosis	29](#_Toc8443)

[Figure S13.Correlation between tissue and serum GDF15 expression.	30](#_Toc20315)

**sMaterials**

**Specific criteria for defining comorbidities**

Advanced comorbid conditions were defined as advanced heart failure (NYHA class III–IV or requiring inotropic/mechanical support), end-stage renal disease (eGFR<15 mL/min/1.73 m² or dialysis dependence), liver cirrhosis with decompensation (ascites, variceal bleeding, or hepatic encephalopathy), and severe active infection requiring hospitalization or meeting sepsis criteria.

**Study population**

After pre-screening according to the inclusion criteria, data from a total of 1462 patients with digestive malignancies and non-cancer controls were included. The digestive malignancies included in this study comprised esophageal cancer (66 cases), gastric cancer (109 cases), hepatocellular carcinoma (141 cases), intrahepatic cholangiocarcinoma (27 cases), hilar cholangiocarcinoma (17 cases), extrahepatic cholangiocarcinoma (17 cases), gallbladder cancer (9 cases), pancreatic cancer (121 cases), periampullary cancer (11 cases), small bowel cancer (5 cases), and colorectal cancer (99 cases).

**Collection of variables**

Clinical predictive variables: This study collected clinical data on patients via the electronic health record system, including (1) Basic information and medical history: age, gender, body mass index (BMI), comorbidities; (2) Laboratory parameters: Complete blood count parameters [red blood cell count (RBC), hemoglobin (Hb), platelet count (PLT)] and C-reactive protein (CRP) measured using the XN-1000 fully automated hematology analyzer (Sysmex, Japan); tumor markers (carcinoembryonic antigen (CEA), carbohydrate antigen 19-9 (CA19-9), Roche Cobas 801, Roche); coagulation parameters (prothrombin time (PT), activated partial thromboplastin time (APTT), D-dimer, fibrinogen (Fbg), Sysmex CS-5100 fully automated analyzer); biochemical parameters (serum alanine aminotransferase (ALT), aspartate aminotransferase (AST), alkaline phosphatase (ALP), γ-glutamyltransferase (GGT), total protein (TP), albumin (ALB), total bilirubin (TBIL), direct bilirubin (DBIL), urea (UREA),estimated glomerular filtration rate (eGFR), Glucose (GLU), B-type natriuretic peptide (BNP), Roche 701 fully automated biochemistry analyzer). All tests are conducted using dedicated reagents compatible with the instruments, and daily quality control results remain within acceptable limits to ensure the reliability of the data.

Tumor-related variables: Through a retrospective analysis, this study collected data on the clinical and pathological characteristics of the patients’ tumours, including clinical staging (according to the 8th edition of the AJCC TNM classification system), primary tumour (T) staging, the maximum diameter of the primary lesion, the size of metastatic lymph nodes, the diameter of distant metastases, the extent of tumour invasion into surrounding tissues or blood vessels and comorbidities (such as chronic kidney disease, atherosclerosis, coronary heart disease)

**Follow-up data**

A total of 622 patients with digestive malignancies were included in the survival analysis. The follow-up coverage rate was 91.5%. The overall median follow-up duration was 216 days. During follow-up, 129 patients were observed to have died or relapsed, accounting for 22.75%.

**Tissue sample collection**

The pathological tissue samples collected included 10 cases each of colorectal cancer, liver cancer, gastric cancer, cholangiocarcinoma, and pancreatic cancer, along with the corresponding normal tissue adjacent to the tumors.

**sMethods**

**Immunohistochemical analysis**

Place the paraffin sections in an oven at 65°C for 1–2 hours, until the paraffin has completely melted. The tissue sections are immediately immersed sequentially in xylene and a gradient of ethanol for dewaxing. The sections were then transferred to ammonium citrate antigen retrieval solution (pH 6.0) (Nanjing Novazene Biotechnology Co., Ltd., Nanjing, catalogue number HC102-01), heated for 5 minutes using high-power microwaves and for 15 minutes using low-power microwaves, and allowed to cool naturally to room temperature. In the dark, immerse the sections in a 3% hydrogen peroxide solution for 10 minutes to inactivate endogenous peroxidase. Next, block the sections with 3% bovine serum albumin (BSA) at 37°C for 20 minutes. After blocking, add GDF15 antibody (Wuhan Sanying Biotechnology Co., Ltd., China, catalog number 27455-1-AP) dropwise at a dilution of 1:200 and incubate at 37°C for 2 hours. Then add the secondary antibody, MaxVision-HRP Mouse/Rabbit Secondary Antibody (Zhongshan Jinqiao Biotechnology Co., Ltd., Beijing, catalogue number KIT5020), and incubate at room temperature for 30 minutes. Staining was performed using DAB as the chromogen, followed by nuclear counterstaining with hematoxylin, 1% hydrochloric acid in ethanol, and 2% ammonium hydroxide. The sections were dehydrated using a gradient of ethanol, air-dried, and mounted with neutral resin.

**H-score method for immunohistochemistry**

The H-score assessment involves two components: staining intensity and the proportion of positive cells. Staining intensity is typically classified under light microscopy as follows: 0 (no staining), 1+ (weak positivity, pale yellow), 2+ (moderate positivity, brownish-yellow), and 3+ (strong positivity, brown). The proportion of cells at each intensity level is then estimated as a percentage of the total number of evaluated cells (usually tumor cells). The final H-score is calculated using a weighted formula: H-score = (percentage of 1+ cells × 1) + (percentage of 2+ cells × 2) + (percentage of 3+ cells × 3).

**SHAP analysis workflow**

To evaluate the interpretability of the top model, we used SHapley Additive exPlanations (SHAP). The feature importance bar plot displays each variable's mean absolute contribution. The SHAP summary (swarm) plot shows the distribution of SHAP values per feature and their link to feature values. The dependence plot visualizes a single feature's association with the prediction, including non-linear effects. For representative cases, waterfall and force plots were made to explain individual predictions by showing each feature's contribution direction and magnitude to the predicted probability.

| Table S1. Summary of missing values in the stratification variables prior to interpolation | | |
| --- | --- | --- |
| Variable | n. missing | % missing |
| Clinical Classification | 0 | 0 |
| Metastatic | 0 | 0 |
| Lymph Node Metastatic | 0 | 0 |
| Metastatic Lesions | 0 | 0 |
| T Category | 0 | 0 |
| Tumor Size | 0 | 0 |
| Multifocal Tumors | 0 | 0 |
| Lymph Node Size | 0 | 0 |
| Size of Metastatic Lesions | 0 | 0 |
| Invasion | 0 | 0 |
| Comorbidities | 0 | 0 |
| Note: With the exception of tumor size, lymph node size, and metastatic lesion size, all other variables relating to digestive malignancies are binary variables. | | |

| Table S2.Summary of MICE multiple imputation settings | |
| --- | --- |
| **Parameter** | **Setting** |
| Imputation method | MICE |
| Number of imputations | 5 |
| Iterations | 20 |
| Seed | 123 |
| Continuous variables | PMM |
| Predictor matrix | All predictors except outcome/ID variables |
| Post-imputation analysis | Subsequent analyses performed using the completed dataset |

| Table S3.characteristics of different feature selection methods | | |
| --- | --- | --- |
| Method | Main advantage | Characteristics in variable selection |
| Univariate logistic regression | Simple and interpretable preliminary screening | Rapidly identifies variables significantly associated with the outcome and reduces dimensionality before multivariate modeling |
| LASSO | Handles high-dimensional data and multicollinearity | Performs coefficient shrinkage and variable selection simultaneously, helping prevent overfitting |
| Boruta algorithm | Robust identification of all relevant features | Based on random forests, it compares real variables with shadow features to retain variables strongly associated with the outcome |
| RF-RFE | Captures nonlinear relationships and interactions | Iteratively removes less important variables using random forest importance rankings to identify the optimal feature subset |
| Note: RF-RFE,Random Forest Recursive Feature Elimination;LASSO,Least Absolute Shrinkage and Selection Operator | | |

| Table S4.Details of the parameters for different machine learning models in the training cohort | | | | |
| --- | --- | --- | --- | --- |
| Rank | Model | AUC | Training Time | Optimal Parameters |
| Training cohort | | | | |
| 1 | Xgboost | 0.887 | 1.6s | nrounds = 276, max_depth = 3, eta = 0.1, gamma = 0.5, colsample_bytree = 0.5, min_child_weight = 1, subsample = 0.6 |
| 2 | Logistic | 0.883 | 1s | parameter = none |
| 3 | SVM | 0.883 | 13.1s | sigma = 0.001, C = 0.09 |
| 4 | NeuralNetwork | 0.855 | 5.6s | size = 6, decay = 0.6 |
| 5 | Adaboost | 0.769 | 9.9s | mfinal = 2, maxdepth = 2, coeflearn = 1 |

| Table S5.Hyperparameter tuning strategy and final selected parameters for the XGBoost model | | |
| --- | --- | --- |
| Hyperparameter | Search range | Final value |
| nrounds | 10–300 | 276 |
| eta | 0.01–0.3 | 0.1 |
| max_depth | 2–5 | 3 |
| subsample | 0.6–0.8 | 0.6 |
| colsample_bytree | 0.4–0.8 | 0.5 |
| gamma | 0–0.5 | 0.5 |
| min_child_weight | 1–5 | 1 |

| Table S6.Detailed information on the main machine learning software packages | | | |
| --- | --- | --- | --- |
| Package | Version number | Function | Application |
| caret | 6.0-94 | createDataPartition() | Randomly divide the derivation cohort into training and internal validation cohorts at a ratio of 7:3 |
|  |  | createFolds() | Split the data into ten folds |
|  |  | nearZeroVar() | Exclude variables with zero variance and near-zero variance |
|  |  | train() | Invoke different algorithms and fine-tune their parameters |
| pROC | 1.18.0 | roc() | Construct ROC curves |
|  |  | auc() | Computes the numeric value of AUC with the trapezoidal rule |
|  |  | ci() | Computes the CI of a ROC curve (Default: the 95% CI are computed with 2000 stratified bootstrap replicates) |
|  |  | coords() | Return the coordinates of ROC curves |
| shapviz | 0.9.0 | shapviz() | Calculate the SHAP values of features |
| DALEX | 2.4.3 | explain() | Encapsulates various machine learning models, provides a unified interface for interpretability analysis |
| xgboost | 1.7.7.1 | xgboost() | Train XGBoost models |
| rmda | 1.6 | decision_curve() | Assessing the value of an intervention (compared with no treatment or intervention) |
| tableone | 0.13.2 | CreateTableOne() | Describe baseline characteristics |
| ResourceSelection | 0.3-6 | hoslem.test() | Hosmer-Lemeshow goodness of fit test |
| boot | 1.3-32 | boot() | Implement non-parametric Bootstrap resampling for statistical inference and model stability verification |
| Note: Abbreviations: XGBoost, eXtreme gradient boosting; SHAP, SHapley Additive exPlanation; ROC, receiver operating characteristic curve; AUC, area under the receiver operating characteristic curve; CI, confidence interval. | | | |

| Table S7.Analysis of characteristics in patients with digestive malignancies and non-cancer control group | | | | | | |
| --- | --- | --- | --- | --- | --- | --- |
|  | Train N=953 | *p*.overall | IV N=409 | *p*.overall | ITC N=100 | *p*.overall |
| group |  |  |  |  |  |  |
| Non-cancer | 517（54.25%） |  | 223（54.52%） |  | 55(55.00%) |  |
| Cancer | 436（45.75%） |  | 186（45.48%） |  | 45(45.00%) |  |
| gender |  |  |  |  |  |  |
| Female | 568 (59.60%) | 0.379 | 229 (55.99%) | 0.284 | 55 (55.00%) | 0.614 |
| Male | 385 (40.40%) |  | 180 (44.01%) |  | 45 (45.00%) |  |
| Age | 53.00 [41.00;62.00] | <0.001 | 50.00 [39.00;60.00] | 0.107 | 52.08 [42.48;58.58] | 0.222 |
| BMI | 21.85 [20.43;23.26] | 0.757 | 21.74 [20.39;22.92] | 0.757 | 21.93 [20.33;23.66] | 0.861 |
| GDF15（pg/mL） | 957.60 [708.83;1326.44] | <0.001 | 970.48 [699.59;1312.21] | <0.001 | 1185.34 [902.12;1502.90] | <0.001 |
| CEA（ng/mL） | 3.78 [2.47;5.62] | <0.001 | 3.80 [2.65;5.47] | <0.001 | 4.48 [3.27;6.56] | <0.001 |
| CA19-9（U/mL） | 38.43 [26.91;54.52] | <0.001 | 37.87 [26.23;54.37] | <0.001 | 52.37 [12.81;139.96] | 0.001 |
| HB（g/L） | 129.98 [122.11;137.24] | 0.826 | 128.74 [121.13;136.03] | 0.53 | 129.28 [123.90;135.01] | 0.925 |
| PLT（×10⁹/L） | 225.00 [174.00;288.00] | 0.016 | 224.00 [172.00;279.00] | 0.522 | 221.43 [192.11;273.18] | 0.16 |
| PT（s） | 12.61 [10.19;15.60] | 0.458 | 12.58 [9.85;15.90] | 0.82 | 13.58 [11.12;16.04] | 0.972 |
| Fbg（g/L） | 3.18 [2.48;3.83] | <0.001 | 3.21 [2.54;3.86] | 0.002 | 3.81 [3.45;4.34] | 0.045 |
| D-Dimer（ng/L） | 450.53 [253.08;689.84] | 0.932 | 473.23 [280.33;703.02] | 0.936 | 1233.32 [305.20;10000.00] | 0.548 |
| ALT（U/L） | 37.84 [23.80;52.91] | 0.578 | 36.21 [23.81;53.73] | 0.434 | 36.72 [26.46;50.91] | 0.19 |
| AST（U/L） | 24.18 [20.20;30.56] | 0.022 | 24.43 [19.86;31.01] | 0.314 | 27.67 [22.67;31.86] | 0.973 |
| ALP（U/L） | 101.11 [43.85;159.75] | <0.001 | 98.49 [43.89;164.38] | <0.001 | 114.10 [63.81;179.95] | 0.097 |
| GGT（U/L） | 33.19 [15.40;50.78] | 0.419 | 31.34 [15.33;48.17] | 0.029 | 38.09 [24.23;54.09] | 0.033 |
| TP（g/L） | 70.92 [66.93;74.89] | 0.388 | 70.91 [66.93;74.28] | 0.707 | 72.01 [69.15;74.23] | 0.362 |
| ALB（g/L） | 43.68 (5.63) | <0.001 | 43.63 (5.73) | <0.001 | 43.54 (6.18) | 0.089 |
| DBIL（μmol/L） | 4.90 [3.20;7.70] | <0.001 | 4.90 [3.10;7.90] | <0.001 | 16.92 [6.88;32.16] | 0.204 |
| UREA（mmol/L） | 5.48 [3.48;8.05] | 0.651 | 5.53 [3.46;7.73] | 0.062 | 5.74 [3.47;7.63] | 0.867 |
| eGFR(mL/min/1.73 m²) | 86.06 [64.81;107.04] | 0.443 | 77.15 [59.02;99.65] | 0.9 | 81.82 [60.66;100.73] | 0.77 |
| BNP(pg/mL) | 12.57 [10.00;25.00] | 0.807 | 12.00 [10.00;22.57] | 0.332 | 12.23 [10.10;22.17] | 0.312 |
| CRP(mg/L) | 13.00 [5.31;22.00] | 0.16 | 12.03 [5.00;22.00] | 0.397 | 12.53 [5.40;22.00] | 0.357 |
| GLU(mmol/L) | 6.10 [5.30;6.80] | 0.688 | 6.00 [5.20;6.70] | 0.537 | 6.20 [5.40;6.80] | 0.517 |
| Comorbidities: |  | 0.914 |  | 0.987 |  | 0.957 |
| No | 864 (90.66%) |  | 367 (89.73%) |  | 88(88.00%) |  |
| Yes | 89 (9.34%) |  | 42 (10.27%) |  | 12(12.00%) |  |
| Note: IV,Internal validation cohort; ITC,Independent test cohort | | | | | | |

| Table S8.Univariate and multivariate regression analyses of clinical predictive factors | | | | |
| --- | --- | --- | --- | --- |
| characteristics | Univariate OR (95%CI) | Univariate *P* | Multivariate OR (95%CI) | Multivariate *P* |
| GDF15 | 1.002(1.002-1.003) | <0.001 | 1.002 (1.002–1.003) | <0.001 |
| Age | 1.016(1.006-1.025) | <0.01 | 1.021 (1.008–1.034) | <0.01 |
| Gender | 0.898(0.694-1.162) | 0.412 |  |  |
| BMI | 1.036(0.987-1.088) | 0.154 |  |  |
| CEA | 1.411(1.316-1.512) | <0.001 | 1.380 (1.269–1.502) | <0.001 |
| CA19-9 | 1.053(1.044-1.062) | <0.001 | 1.055 (1.043–1.066) | <0.001 |
| HB | 1.001(0.984-1.017) | 0.939 |  |  |
| PLT | 1.002(1-1.003) | <0.05 |  |  |
| Fbg | 1.5(1.328-1.696) | <0.001 | 1.385 (1.179–1.627) | <0.001 |
| D-Dimer | 1(0.999-1) | 0.32 |  |  |
| ALT | 1.004(0.999-1.01) | 0.137 |  |  |
| ALP | 1.007(1.005-1.009) | <0.001 | 1.005 (1.003–1.007) | <0.001 |
| AST | 1.009(0.994-1.025) | 0.234 |  |  |
| GGT | 0.999(0.994-1.004) | 0.807 |  |  |
| ALB | 1.027(1.003-1.051) | <0.05 |  |  |
| DBIL | 1.013(1.008-1.019) | <0.001 | 1.007 (1.002–1.012) | <0.01 |
| eGFR | 0.995(0.991-1) | <0.05 |  |  |
| BNP | 1.004(0.997-1.011) | 0.281 |  |  |
| TP | 0.978(0.949-1.009) | 0.164 |  |  |
| CRP | 1.005(0.998-1.012) | 0.142 |  |  |
| GLU | 1.057(0.918-1.218) | 0.438 |  |  |
| Comorbidities | 1.268(0.819-1.964) | 0.287 |  |  |
| PT | 1.004(0.971-1.038) | 0.818 |  |  |
| Note:OR,Odds Ratio;BMI,Body Mass Index | | | | |

| Table S9.All regression coefficients | |
| --- | --- |
| Variable | Coefficients |
| (Intercept) | -9.022625 |
| GDF15 | 0.001878 |
| Age | 0.019472 |
| CA19-9 | 0.05215 |
| CEA | 0.340313 |
| Fbg | 0.456818 |
| ALP | 0.005004 |
| DBIL | 0.007199 |

| Table S10.AUC values for different cancer types in the XGBoost model on the training and validation cohorts. | | |
| --- | --- | --- |
| Cancer type | Case (n) | AUC(95% CI) |
| Training cohort | | |
| Esophageal cancer | 46 | 0.862(0.785–0.924) |
| Gastric cancer | 75 | 0.868(0.816–0.914) |
| Hepatocellular carcinoma | 99 | 0.91(0.879–0.940) |
| Cholangiocarcinoma | 43 | 0.893(0.841–0.940) |
| Pancreatic cancer | 85 | 0.886(0.846–0.923) |
| Colorectal cancer | 69 | 0.888(0.847–0.924) |
| Internal Validation cohort | | |
| Esophageal cancer | 20 | 0.993(0.983–0.999) |
| Gastric cancer | 34 | 0.971(0.949–0.988) |
| Hepatocellular carcinoma | 42 | 0.918(0.872–0.955) |
| Cholangiocarcinoma | 18 | 0.894(0.828–0.950) |
| Pancreatic cancer | 36 | 0.855(0.798–0.907) |
| Colorectal cancer | 30 | 0.743(0.649–0.831) |

| Table S11.The Hosmer-Lemeshaux test for assessing calibration \| \| \| \|; Group \| X-squared \| df \| p-value \| Brier; Training cohort \| 6.8314 \| 8 \| 0.5549 \| 0.144; Internal Validation cohort \| 11.287 \| 8 \| 0.186 \| 0.114; Independent test cohort \| 87.345 \| 8.00 \| 0.00 \| 0.22 Table S12.Performance metrics of five machine learning models on the training and validation cohort | | | | | | | | |
| --- | --- | --- | --- | --- | --- | --- | --- | --- |
| Model | AUC | Accuracy | Sensitivity | Specificity | Precision | PPV | NPV | F1 Score |
| Training cohort | | | | | | | | |
| Logistic | 0.887（0.866-0.908） | 0.815 | 0.789 | 0.838 | 0.804 | 0.804 | 0.799 | 0.796 |
| SVM | 0.885（0.864-0.906） | 0.813 | 0.761 | 0.857 | 0.818 | 0.818 | 0.782 | 0.789 |
| NeuralNetwork | 0.894（0.874-0.914） | 0.829 | 0.768 | 0.88 | 0.844 | 0.844 | 0.791 | 0.804 |
| Xgboost | 0.917（0.9-0.934） | 0.836 | 0.81 | 0.859 | 0.829 | 0.829 | 0.819 | 0.819 |
| Adaboost | 0.799（0.773-0.826） | 0.747 | 0.837 | 0.671 | 0.682 | 0.682 | 0.805 | 0.752 |
| Internal Validation cohort | | | | | | | | |
| Logistic | 0.88（0.847-0.912） | 0.797 | 0.763 | 0.825 | 0.785 | 0.785 | 0.777 | 0.774 |
| SVM | 0.879（0.847-0.912） | 0.802 | 0.72 | 0.87 | 0.822 | 0.822 | 0.757 | 0.768 |
| NeuralNetwork | 0.884（0.853-0.915） | 0.8 | 0.737 | 0.852 | 0.806 | 0.806 | 0.764 | 0.77 |
| Xgboost | 0.891（0.86-0.922） | 0.819 | 0.876 | 0.771 | 0.762 | 0.762 | 0.861 | 0.815 |
| Adaboost | 0.769（0.726-0.813） | 0.738 | 0.796 | 0.691 | 0.682 | 0.682 | 0.772 | 0.734 |
| Independent Test cohort | | | | | | | | |
| Logistic | 0.762(0.666-0.858) | 0.78 | 0.911 | 0.673 | 0.695 | 0.736 | 0.879 | 0.788 |
| SVM | 0.754(0.655-0.852) | 0.78 | 0.911 | 0.673 | 0.695 | 0.736 | 0.879 | 0.788 |
| NeuralNetwork | 0.762(0.661-0.862) | 0.78 | 0.933 | 0.655 | 0.689 | 0.73 | 0.902 | 0.792 |
| Xgboost | 0.886(0.822-0.949) | 0.81 | 0.844 | 0.782 | 0.76 | 0.795 | 0.833 | 0.8 |
| Adaboost | 0.748(0.657-0.840) | 0.75 | 1 | 0.545 | 0.643 | 0.687 | 1 | 0.783 |
| Note: Logit, Logistic Regression; SVM, Support Vector Machine; XGBoost, Extreme Gradient Boosting;Adaboost，Adaptive Boosting； PPV: positive predictive value; NPV: negative predictive value; AUC: the area under the receiver operating characteristic (ROC) curve | | | | | | | | |

| Table S13. Subgroup performance of the seven-variable model | | | | | |
| --- | --- | --- | --- | --- | --- |
| Malignancy subgroup | N | AUROC | 95% CI | Sensitivity | Specificity |
| Esophageal cancer | 66 | 0.838 | 0.780–0.896 | 0.742 | 0.855 |
| Gastric cancer | 109 | 0.878 | 0.844–0.913 | 0.789 | 0.809 |
| Colorectal cancer | 99 | 0.876 | 0.843–0.910 | 0.788 | 0.799 |
| Hepatobiliary malignancies | 210 | 0.904 | 0.878–0.929 | 0.814 | 0.858 |
| Pancreatic malignancies | 132 | 0.907 | 0.878–0.936 | 0.811 | 0.858 |
| Note: Due to the limited sample size of several individual tumor subtypes, malignancies were grouped according to their primary anatomical origin. Hepatobiliary malignancies comprised hepatocellular carcinoma, intrahepatic cholangiocarcinoma, hilar cholangiocarcinoma, extrahepatic cholangiocarcinoma, and gallbladder cancer. Pancreatic malignancies comprised pancreatic cancer and periampullary cancer. | | | | | |

| Table S14.Multicollinearity analysis of variables included in Cox regression | | | | | | | |
| --- | --- | --- | --- | --- | --- | --- | --- |
| Variable | Unstandardized Coefficients | Std. Error | Standardized Coefficients | t | *P* | CollinearityStatistics | |
|  | B |  | Beta |  |  | Tolerance | VIF |
| (Constant) | 201.831 | 15.927 |  | 12.672 | 0.001 |  |  |
| Clinical Classification | -6.861 | 9.202 | -0.033 | -0.746 | 0.456 | 0.886 | 1.129 |
| Lymph Node Metastatic | 4.291 | 16.063 | 0.022 | 0.267 | 0.789 | 0.27 | 3.698 |
| GDF15 | -5.632 | 8.358 | -0.028 | -0.674 | 0.501 | 0.992 | 1.009 |
| Distant Metastatic | 15.276 | 11.78 | 0.07 | 1.297 | 0.195 | 0.597 | 1.675 |
| Gender | 2.514 | 9.417 | 0.013 | 0.267 | 0.79 | 0.795 | 1.258 |
| Metastatic | -33.967 | 18.697 | -0.16 | -1.817 | 0.07 | 0.225 | 4.446 |

| Table S15.Univariate and multivariate Cox regression | | | | | | | | | | | |
| --- | --- | --- | --- | --- | --- | --- | --- | --- | --- | --- | --- |
| Variables | Univariate | | | | |  | Multivariate | | | | |
|  | β | S.E | Z | *P* | HR (95%CI) |  | β | S.E | Z | *P* | HR (95%CI) |
| GDF15 |  |  |  |  |  |  |  |  |  |  |  |
| Low |  |  |  |  | 1.00 (Reference) |  |  |  |  |  | 1.00 (Reference) |
| High | 0.95 | 0.21 | 4.61 | 0.001 | 2.59 (1.73 ~ 3.87) |  | 0.99 | 0.21 | 4.75 | 0.001 | 2.68 (1.78 ~ 4.03) |
| Clinical Classification |  |  |  |  |  |  |  |  |  |  |  |
| Ⅰ-Ⅱ |  |  |  |  | 1.00 (Reference) |  |  |  |  |  | 1.00 (Reference) |
| Ⅲ-Ⅳ | 0.32 | 0.18 | 1.79 | 0.073 | 1.38 (0.97 ~ 1.96) |  | 0.46 | 0.19 | 2.41 | 0.016 | 1.59 (1.09 ~ 2.32) |
| Lymph Node Metastatic |  |  |  |  |  |  |  |  |  |  |  |
| No |  |  |  |  | 1.00 (Reference) |  |  |  |  |  | 1.00 (Reference) |
| Yes | 0.61 | 0.19 | 3.21 | 0.001 | 1.85 (1.27 ~ 2.69) |  | 0.05 | 0.4 | 0.11 | 0.91 | 1.05 (0.48 ~ 2.29) |
| Distant Metastatic |  |  |  |  |  |  |  |  |  |  |  |
| No |  |  |  |  | 1.00 (Reference) |  |  |  |  |  | 1.00 (Reference) |
| Yes | -0.43 | 0.22 | -1.96 | 0.049 | 0.65 (0.43 ~ 0.99) |  | -0.67 | 0.28 | -2.41 | 0.016 | 0.51 (0.30 ~ 0.88) |
| Gender |  |  |  |  |  |  |  |  |  |  |  |
| Female |  |  |  |  | 1.00 (Reference) |  |  |  |  |  | 1.00 (Reference) |
| Male | -0.04 | 0.18 | -0.2 | 0.844 | 0.96 (0.67 ~ 1.38) |  | 0.02 | 0.2 | 0.11 | 0.914 | 1.02 (0.69 ~ 1.52) |
| Metastatic |  |  |  |  |  |  |  |  |  |  |  |
| No |  |  |  |  | 1.00 (Reference) |  |  |  |  |  |  |
| Yes | 0.59 | 0.21 | 2.84 | 0.005 | 1.81 (1.20 ~ 2.73) |  |  |  |  |  |  |
| Note:HR: Hazards Ratio, CI: Confidence Interval.Due to clinical overlap among lymph node metastasis, distant metastasis, and metastasis status, only representative variables were included in the final multivariable Cox model. | | | | | | | | | | | |

**Figure S1.**Overview of missing data distribution for included clinical variables. (A) Missing rates for all variables. The x-axis represents the percentage of missing data for each variable; the y-axis represents the various clinical indicators included in the study. The length of the grey horizontal bars corresponds to the overall missing rate for the respective indicators. (B) Missing probability plot, where the vertical axis represents the proportion of samples for different missing data patterns; the horizontal axis shows the names of the clinical variables. The height of the red bars indicates the frequency of that missing data pattern across the entire sample

**Figure S2.** Methods for selecting variables based on different criteria. (A) Variable selection using univariate analysis. (B-C) Demonstration of the LASSO feature selection method. (D-E) Demonstration of the Boruta feature selection method. (F) Demonstration of the RF-RFE feature selection method. (G) Correlations between all variables. (H) VIF of candidate predictor variables. (I) Intersection of the four feature selection methods.

**Figure S3.**Performance and Evaluation of Five Models.(A), (C), and (E) show the DCA curves for the training cohort, internal validation cohort, and independent test cohort, respectively. (B), (D), and (F) show the calibration curves for the training cohort, internal validation cohort, and independent test cohort, respectively.

**Figure S4.** Confusion matrices for five machine learning models in the internal validation. (A–E, G–K) Confusion matrices for XGBoost, support vector machines (SVM), neural networks, logistic regression, and AdaBoost models. This diagram includes true positives, true negatives, false positives, and false negatives. (F) A radar chart showing comprehensive performance indicators, including AUC, accuracy, sensitivity, specificity, precision, and F1 score.

**Figure S5.**10-fold cross-validation and 100 bootstraps. (A) XGBoost 10-fold cross-validation ROC curve. (B) The probability distribution of predictions from the XGBoost model under 10-fold cross-validation. The blue region represents the probability distribution of predictions for non-cancer samples, while the red region represents the probability distribution of predictions for cancer samples. (C) Precision-Recall (PR) curves of the XGBoost model in 10-fold cross-validation. The mean area under the PR curve (AUPRC) is 0.965. (E) Feature importance of the XGBoost model in 10-fold cross-validation, with 95% confidence intervals (CI). Features are sorted by mean gain in descending order. (E) ROC curve of the model. The red solid line represents the mean ROC curve, with an AUC of 0.900 (95% CI: 0.871–0.926), and the blue thin lines represent 100 bootstrap curves. (F) Histogram of AUC distribution from 100 bootstrap resamplings, with the blue dashed line indicating the mean AUC. (G) Calibration curve of the model. The red solid line represents the mean calibration curve, the gray dashed line represents the ideal line, and the blue thin lines represent 100 Bootstrap curves. (H) Bootstrap-corrected decision curve analysis. The blue solid line represents the net benefit of the model, the gray dashed line represents the treat-all strategy, and the gray dotted line represents the treat-none strategy. AUC, area under the receiver operating characteristic curve; CI, confidence interval.

**Figure S6.**Evaluation of clinical net benefit, and calibration of different prediction models (training cohort vs. internal validation cohort). (A,C) Decision curve analysis (DCA) of the models in the training cohort and internal validation cohort, respectively. (B,D) Calibration curves of the models in the training cohort and internal validation cohort, respectively.

**Figure S7.**SHAP Summary Analysis and Model Performance Evaluation. (A) SHAP dependence plots for key predictive features. (B-C) Radar plots of model performance metrics in the internal and independent test cohort.

**Figure S8.**Evaluation of the model’s predictive performance across different clinical progression strata. (A) ROC curve for clinical classification. (B) Inter-group differences in the model’s predicted probability of clinical staging. (C) ROC curve for lymph node metastasis (D) Inter-group differences in the model’s predicted probability of lymph node metastasis. (E) ROC curve for the classification of distant metastasis. (F) Inter-group differences in the model’s predicted probability of distant metastasis.

**Figure S9.**AnalysisS6. Analysis of ROC curves for tumor markers in corresponding negative populations. (A) ROC curve of GDF15 in CA19-9 negative population. (B) ROC curve of GDF15 in CEA-negative population. (C) ROC curve of GDF15 in patients with both negative CA19-9 and CEA. (D) ROC curve of CEA in CA19-9 negative population. (E) ROC curve of CA19-9 in CEA-negative population.

**Figure S10.**Age Correlation and Stratified Predictive Performance of GDF15. (A)Correlation heatmap of clinical features. (B)ROC curves of GDF15 across different age subgroups. (C)ROC curve of GDF15 for predicting comorbidities in digestive malignancies. (D)Forest plot from logistic regression for Age, eGFR and GDF15 related to comorbidities.

**Figure S11.**Risk stratification and prediction chart for malnutrition in digestive malignancies. (A) Risk stratification distribution of patients in the training cohort. (B) Risk stratification distribution of patients in the validation cohort. Note: The main plot curve represents the predicted risk scores for patients generated by the XGBoost model, sorted from lowest to highest score; the colored background sections denote low, medium, and high-risk groups based on model scores; the bottom bars indicate the patients’ risk stratification status as defined by the group(cancer and non-cancer). (C)A clinical nomogram was constructed based on the predictive model, which could individually predict the probability of digestive malignancies for each patient by summing the scores of each variable and converting the total score to the corresponding predicted risk.

**Figure S12.** Association of Clinical Characteristics with Patient Survival Prognosis. (A) Survival curves of patients with different GDF15 levels. (B) Survival curves stratified by clinical stage. (C) Survival curves stratified by lymph node metastasis status. (D) Survival curves stratified by distant metastasis status. (E) Survival curves stratified by overall metastasis status. (F) Survival curves stratified by gender. All HR values presented are univariate hazard ratios.

**Figure S13.**Correlation between tissue and serum GDF15 expression.
